# Seminal extracellular vesicles subsets modulate gene expression in cumulus cells of porcine in vitro matured oocytes

**DOI:** 10.1038/s41598-022-22004-7

**Published:** 2022-11-09

**Authors:** Yentel Mateo-Otero, Marc Yeste, Jordi Roca, Marc Llavanera, Diego Bucci, Giovanna Galeati, Marcella Spinaci, Isabel Barranco

**Affiliations:** 1 Unit of Cell Biology, Department of Biology, Faculty of Sciences, University of Girona, S17003 Girona, Spain; 2 Biotechnology of Animal and Human Reproduction (TechnoSperm), Institute of Food and Agricultural Technology, University of Girona, S17003 Girona, Spain; 3 Catalan Institution for Research and Advanced Studies (ICREA), S08010 Barcelona, Spain; 4 Department of Medicine and Animal Surgery, Faculty of Veterinary Medicine, International Excellence Campus for Higher Education and Research “Campus Mare Nostrum”, University of Murcia, S30100 Murcia, Spain; 5 Department of Veterinary Medical Sciences, University of Bologna, Via Tolara di Sopra 50, Ozzano dell’Emilia, IT40064 Bologna, Italy

**Keywords:** Animal physiology, Reproductive biology

## Abstract

Seminal plasma (SP), a fluid composed mainly by secretions from accessory sex glands, contains a heterogenous population of extracellular vesicles (EVs), involved in several reproductive physiological processes. Seminal plasma has been found to modulate ovary function, in terms of hormone secretion and immune regulation. This study evaluated the potential effect of SP-EV-subsets on the modulation of cumulus-oocyte-complex (COCs) physiology during in vitro maturation (IVM). Two SP-EV-subsets, small-EVs (S-EVs) and large-EVs (L-EVs), were isolated from pig SP by size-exclusion-chromatography. Next, COCs were IVM in the absence (control) or presence of each SP-EV-subset to evaluate their uptake by COCs (PKH67-EVs labelling) and their effect on oocyte and cumulus cells (CCs) (gene expression, and progesterone and estradiol-17β levels). S-EVs and L-EVs were able to bind CCs but not oocytes. Supplementation with L-EVs induced changes (*P* ≤ 0.05) in the transcript levels of oocyte maturation- (*HAS2*) and steroidogenesis-related genes (*CYP11A1* and *HSD3B1*) in CCs. No effect on nuclear oocyte maturation and progesterone and estradiol-17β levels was observed when COCs were IVM with any of the two SP-EV-subsets. In conclusion, while SP-EV-subsets can be integrated by CCs during IVM, they do not affect oocyte maturation and only L-EVs are able to modulate CCs function, mainly modifying the expression of steroidogenesis-related genes.

## Introduction

Intercellular communication is critical for the coordination of individual cells behaviour in multicellular organisms^1^. Extracellular vesicles (EVs), a heterogeneous collection of nanosized membrane-enclosed vesicles released from most cells, have emerged as novel intercellular mediators^2^. These EVs play an essential role in physiological and pathological processes^3,4^, including those related to reproduction^5^. The EVs cargo (proteins, lipids, metabolites and nucleic acids) can be transferred into targeted cells modulating their function^6,7^. Indeed, the EV-cargo seems to be highly dependent on cell source, physiologic conditions, and releasing mechanism^8^. In this sense, depending on their biogenesis pathway and size, EVs released by healthy living cells can be categorised into two subsets: (i) exosomes (small EVs, ~ 40 to 200 nm; endosomal origin); and, (ii) microvesicles (large EVs, ~ 200–1000 nm; plasma membrane origin)^9^. These EVs-subsets display a different composition, suggesting a distinct biological function^10–13^.

Extracellular vesicles have been found in a wide range of reproductive fluids including seminal plasma (SP), a fluid composed mainly by secretions of male accessory sex glands^14^. Mounting evidence indicates that SP promotes reproductive success through the modulation of sperm function^15,16^ and the regulation of the immune environment in the female reproductive tract after mating or insemination^17–19^. Because SP contains a large diversity of EV-subsets^20–23^, one may suggest that they could account for some of the roles attributed to this fluid^24^. While SP-EVs have been reported to modulate sperm functional processes, such as sperm maturation, motility, capacitation, and sperm-zona pellucida binding^25–35^, studies assessing the role of SP-EVs on female reproductive physiology are still scarce. Remarkably, SP-EVs have been proven to modulate endometrial immune and inflammatory response^36–38^ and to enhance decidualisation^39^.

Evidence supports that SP may also affect ovarian function. Some meta-analyses in humans suggested that intravaginal or intracervical SP infusion at the time of oocyte pick-up improves pregnancy rates^40,41^. In porcine, uterine exposure to SP was observed to: (i) increase plasma progesterone (P4) and make preovulatory follicles more responsive to growth factors and gonadotrophin-stimulated cell proliferation^42^; (ii) modulate the immune-cytokine network in ovaries and positively regulate oocyte maturation^43^, and (iii) accelerate the ovulation process^44^. These insights may contribute to explain why, as observed in bovine^45^ and porcine^17,46,47^, priming the uterus with SP affects gene expression in the embryo and enhances its development. Although the molecular mechanisms underlying these findings are yet to be reported, SP-EVs may hold the key. Given the essential role played by both SP and EVs on gamete–female reproductive tract crosstalk^5,48^, addressing the role of SP-EVs in this dialog seems imperative. To date, only an in vitro study conducted in rabbit has demonstrated that EVs isolated from cell cultures of testis, epididymis and prostate are able to modulate cumulus cells (CCs) function during in vitro maturation (IVM)^49^. Cumulus cells play an important role during nuclear and cytoplasm maturation of the oocyte, interacting with the female gamete through gap junctions and paracrine factors^50,51^. Yet, the interaction between EVs isolated from SP and female gametes has not been investigated.

The present study aimed to (1) isolate two EV-subsets, small-EV (S-EV) and large-EV (L-EV) from SP, (2) analyse whether cumulus oocyte complexes (COCs) are able to interact with each of these SP-EV subsets, and (3) investigate the effect of each SP-EV subset on oocyte nuclear maturation and CCs during IVM, in terms of gene expression and steroid synthesis. The results showed herein could also be of interest for humans, as the porcine species is a valuable experimental model for human reproductive medicine^52^.

## Results

### Characterisation of SP-EV subsets

**Figure 1:**
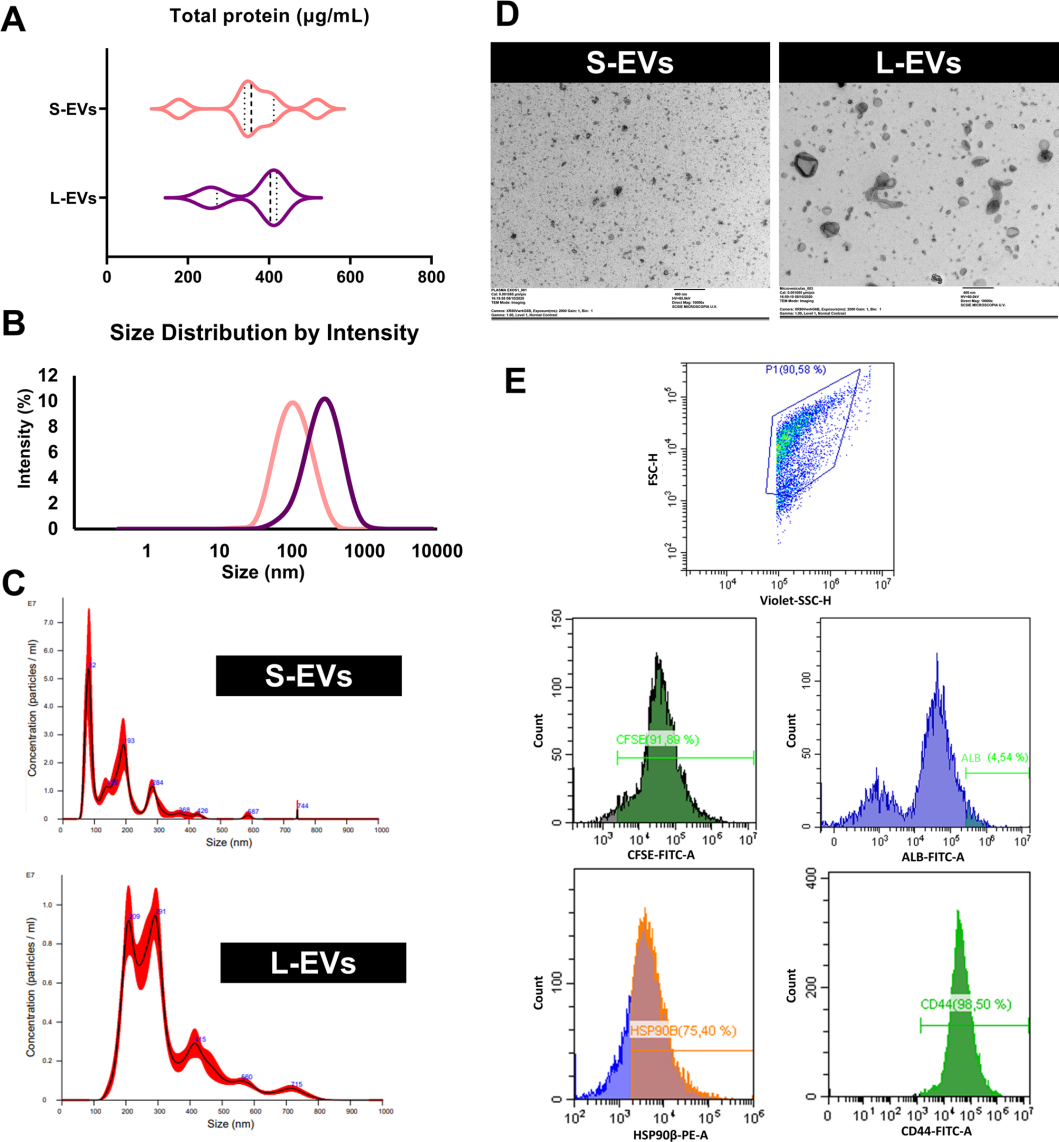
Characterisation of small- (S-EV) and large-extracellular vesicles (L-EV) isolated from seminal plasma samples (SP; *n* = 7) using Size Exclusion Chromatography (SEC). (**A**) Violin plots represent total protein concentration levels and distribution in the two SP-EV subsets (pink: S-EVs; purple: L-EVs). Dashed line represents the median and dotted lines the 25–75% quartiles. This figure was created using GraphPad Prism version 8.2.1 (GraphPad Software, Inc., La Jolla, CA, USA; https://www.graphpad.com/). (**B**) Particle size distribution of the two SP-EV subsets assessed by dynamic light scattering **(DLS).** Each curve represents an average of intensity size distributions of all samples for S-EVs (pink) and L-EVs (purple). (**C**) Representative histogram of particle size distribution of the two SP-EV subsets assessed by nanoparticle tracking analysis (NTA). (**D**) Morphology and size of SP-EV subsets using transmission electron microscopy (TEM). (**E**) Flow cytometry analysis of SP-EV subsets. Representative histogram of CFSE/CD44/HSP90β/ALB expression in the two EV-subsets. CFSE: Carboxyfluorescein succinimidyl ester; ALB: Albumin.

Figure 1 summarizes the characterisation of each SP-EVs subset. The concentration (μg/mL) of total protein was similar between S-EV (mean ± SD; 361.5 ± 101.9) and L-EV (367.5 ± 77.33) samples. Size-distribution was evaluated by dynamic light scattering (DLS), nanoparticle tracking analysis (NTA) and transmission electron microscopy (TEM), revealing that S-EV and L-EV samples were enriched in small and large EVs (Fig. 1A). DLS analysis revealed differences in size-distribution (*P* ≤ 0.001) between the two EV-samples, as EVs were smaller in S-EV (mean ± SD; 118.4 ± 8.99 nm) than in L-EV specimens (303.9 ± 15.86 nm) (Fig. 1B). These results were further confirmed by NTA, which showed that the EV-diameter was greater in L-EV samples (mode ± SD; 257.91 ± 97.26 nm) than in S-EV samples (167.09 ± 89.46 nm) (Fig. 1C). NTA also demonstrated that particle concentration (mean ± SD) did not differ between S-EV and L-EV samples (1.50 × 10^9^ ± 2.39 × 10^8^ *vs* 1.89 × 10^9^ ± 1.36 × 10^8^, respectively). TEM (Fig. 1D) revealed that EVs of S-EV samples corresponded to a population of membrane-enclosed structures with a relatively homogeneous size ranging between ~ 30 and 100 nm in diameter. In contrast, EVs from L-EV samples comprised a more heterogeneous population with diameters ranging from ~ 100 to 350 nm. The TEM images also revealed that most EVs had a dense appearance and a rounded shape. Flow cytometry analysis (Fig. 1E) was based on the physical properties of EVs, as evaluated by Forward scatter (FSC) and Violet-Side Scatter (SSC-A). First, to distinguish intact EVs from membrane fragments and electronic noise, events in the EV gate were discriminated by CFSE-labelling. The percentages (mean ± SD) of CFSE-positive events were 83.96 ± 2.36% and 73.81 ± 6.57% for S-EV and L-EV samples, respectively. In S-EV samples, the percentages of EVs positive to CD44 and heat shock protein 90-β (HSP90β) were 97.88 ± 0.47% and 86.60 ± 7.18%, respectively. Similarly, in L-EV samples, the percentages of EVs positive to CD44 and HSP90β were 97.78 ± 0.43% and 88.13 ± 6.50%, respectively. Flow cytometry results confirmed a high-purity EV enrichment in the two EV-samples, as the percentages of albumin were 4.19 ± 0.53% and 4.14 ± 1.14% in S-EV and L-EV samples, respectively.

### CCs, but not oocytes, are able to bind both SP-EV subsets

**Figure 2:**
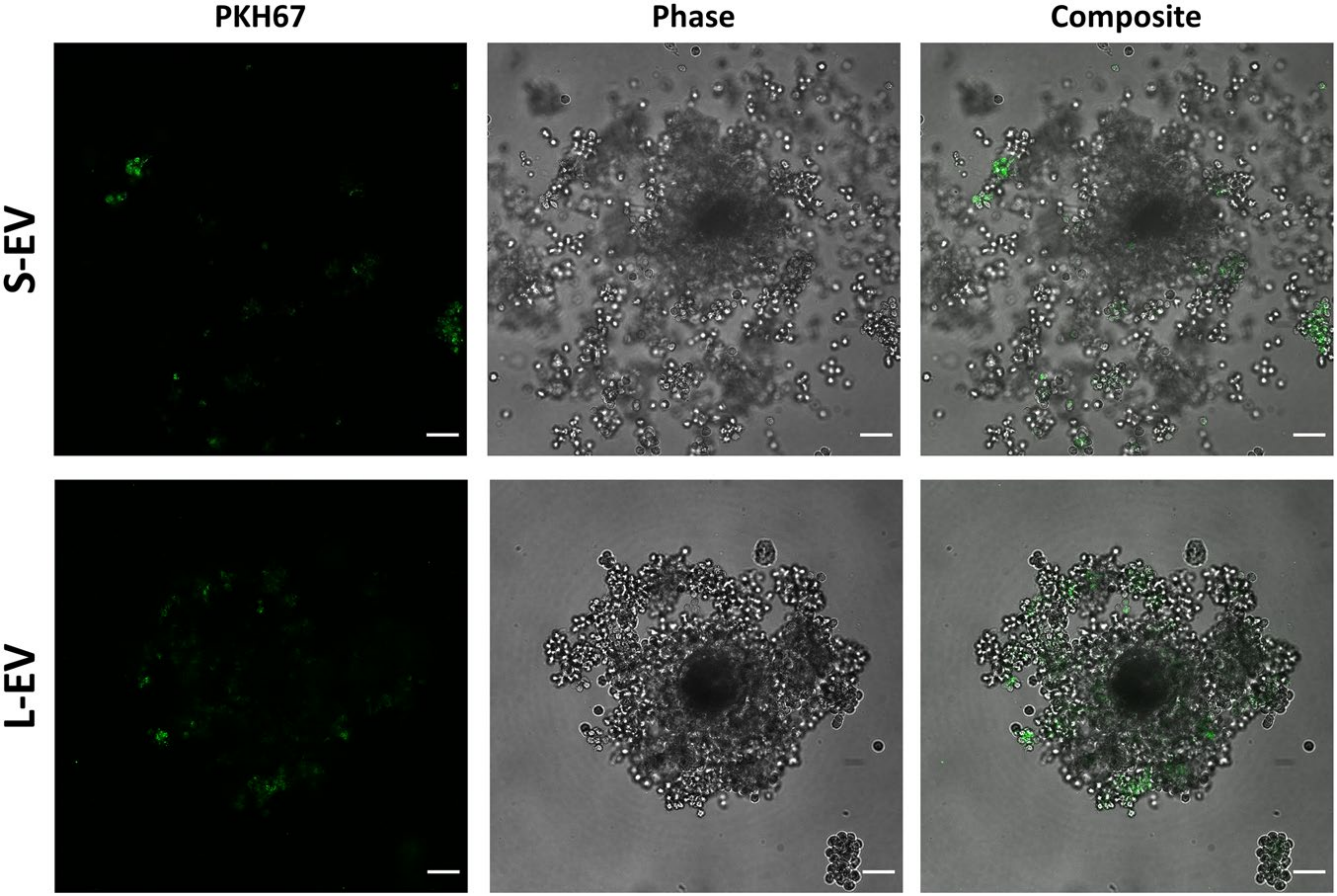
Uptake of seminal plasma extracellular vesicles (SP-EV) by COCs during in vitro maturation (IVM). Representative figures of the uptake of small- (S-EV) and large-EV (L-EV) subsets by COCs. SP-EVs were stained using PKH67 and added to medium for the whole IVM process. Scale bar 50 µm.

The uptake of SP-EVs by COCs was assessed at the end of IVM following the procedures described below (Methods; Sects. “SP-EVs labelling” about SP-EVs labelling, and "Experimental Design" about the experimental design). The presence of the two labelled SP-EVs (S-EVs and L-EVs) was confirmed in the plasma membrane of CCs as green-fluorescent spots, but not in oocytes (Fig. 2). No green-fluorescent spots were found in the negative control (COCs incubated with PKH67–PBS).

### Supplementation with either of the two SP-EV subsets during IVM does not affect oocyte nuclear maturation

The putative effect of the two SP-EV subsets on oocyte nuclear maturation was assessed at the end of IVM (Methods; Sect. “Experimental design” about the experimental design). The percentage of oocytes reaching the metaphase II (MII) stage at the end of IVM was similar (*P* ≥ 0.05) between those matured in the presence of SP-EVs (S-EVs and L-EVs) and the control (no SP-EVs): control: 100.00 ± 0.00%, S-EV low: 97.27 ± 5.04%, S-EV high: 99.59 ± 4.15%, L-EV low: 98.13 ± 4.46%, and L-EV high: 98.74 ± 4.79% (data normalised against the control of the same oocyte batch; the percentage of oocytes reaching the MII stage in the control group was [mean ± SD] 97.8 ± 2.4).

### Adding any of the two SP-EV subsets during IVM modifies CCs gene expression

**Figure 3:**
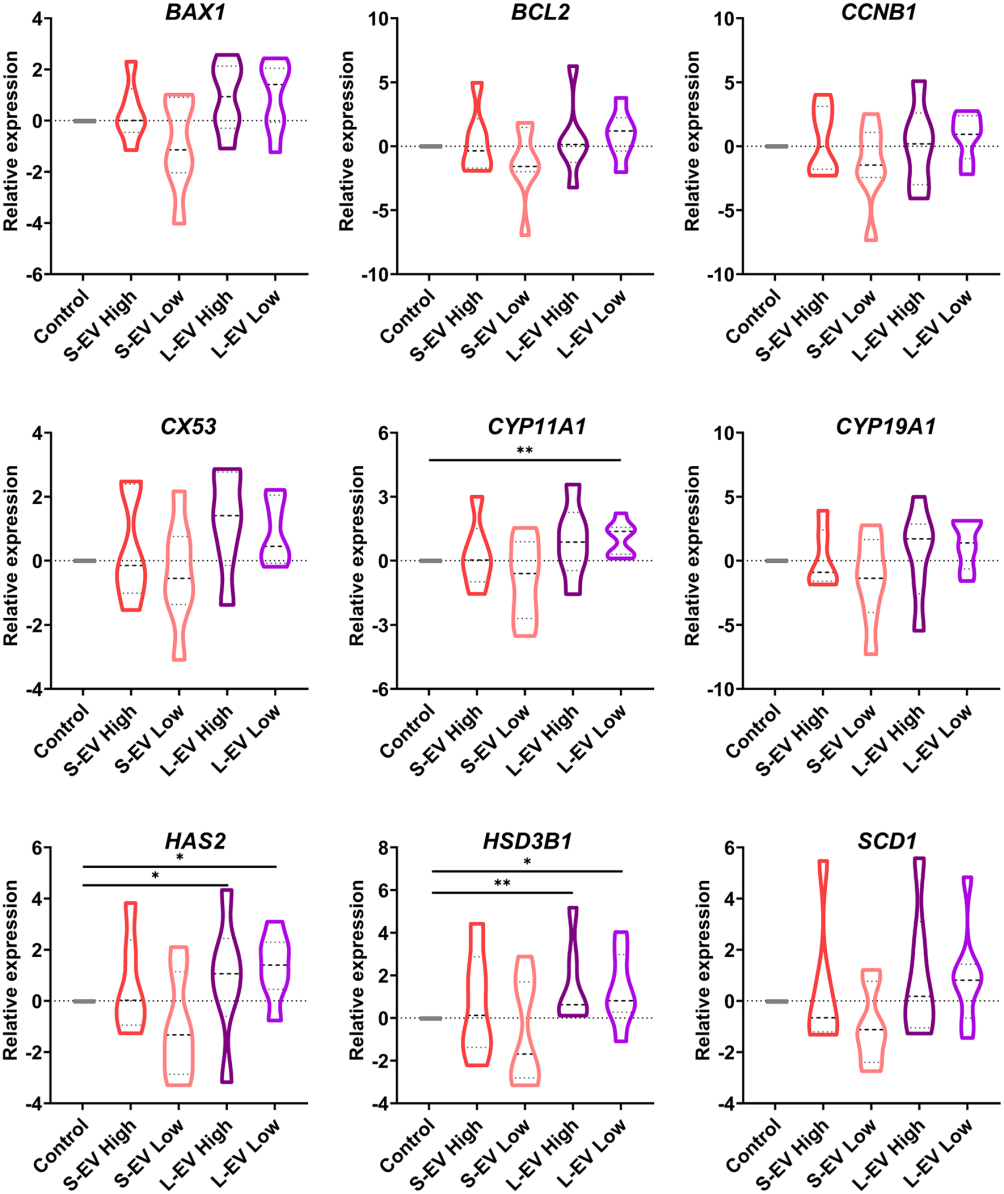
Violin plots representing relative expression levels of *BAX1*, *BCL2*, *CCNB1*, *CX53*, *CYP11A1*, *CYP19A1*, *HAS2*, *HSD3B1* and *SCD1* in cumulus cells in response to the presence of small- (SP-EV) or large-extracellular vesicles (L-EV) at two concentrations (Protein concentration; Low: 0.1 mg/mL; High: 0.2 mg/mL) during in vitro maturation (IVM). Data are represented as ∆∆Ct (calculated with the Livak method, using *RPL19* as housekeeping gene). Dashed line represents the median and dotted lines the 25–75% quartiles. Differences are indicated as (*) for *P* ≤ 0.05 and (**) for *P* ≤ 0.001. This figure was created using GraphPad Prism version 8.2.1 (GraphPad Software, Inc., La Jolla, CA, USA; https://www.graphpad.com/).

The relative abundance of transcripts from nine candidate genes involved in four signalling pathways was assessed: (i) cell apoptosis (*B-cell lymphoma 2* (*BCL2*) and *BCL2 Associated X* (*BAX));* (ii) cell proliferation (*Cyclin B1* (*CCNB1))*; (iii) oocyte maturation (*Connexin 43* (*CX43*), *Hyaluronan Synthase 2* (*HAS2*) and *Stearoyl-CoA desaturase 1* (*SCD1*)); and (iv) steroidogenesis (*Cytochrome P450 Family 11 Subfamily A Member 1* (*CYP11A1*), *Hydroxy-Delta-5-Steroid Dehydrogenase 3 Beta* (*HSD3B1*) and *aromatase* (*CYP19A1*) (Supplementary File 1). Only one of the genes related to oocyte maturation was altered in response to SP-EVs. Specifically, the expression levels of *HAS2* were higher (*P* ≤ 0.05) in CCs supplemented with L-EVs at both concentrations compared to the control (high concentration: 0.89 ± 2.46 *vs*. 0.00 ± 0.00; low concentration: 1.34 ± 1.28 *vs*. 0.00 ± 0.00). In addition, the expression levels of steroidogenesis genes, specifically *CYP11A1* and *HSD3B1*, differed (*P* ≤ 0.05) between CCs supplemented with L-EVs and the control (Fig. 3). *CYP11A1* expression was higher in CCs supplemented with L-EVs at low-concentration compared to the control (1.05 ± 0.80 *vs*. 0.00 ± 0.00, *P* ≤ 0.001). Regarding *HSD3B1*, its expression levels were higher in CCs supplemented with L-EVs at both concentrations than the control (high concentration: 1.56 ± 2.00 *vs*. 0.00 ± 0.00, *P* ≤ 0.001; low concentration: 1.34 ± 1.72 *vs*. 0.00 ± 0.00, *P* ≤ 0.05).

### Supplementation of the IVM medium with SP-EVs does not affect CCs progesterone and estradiol-17β secretion

**Figure 4:**
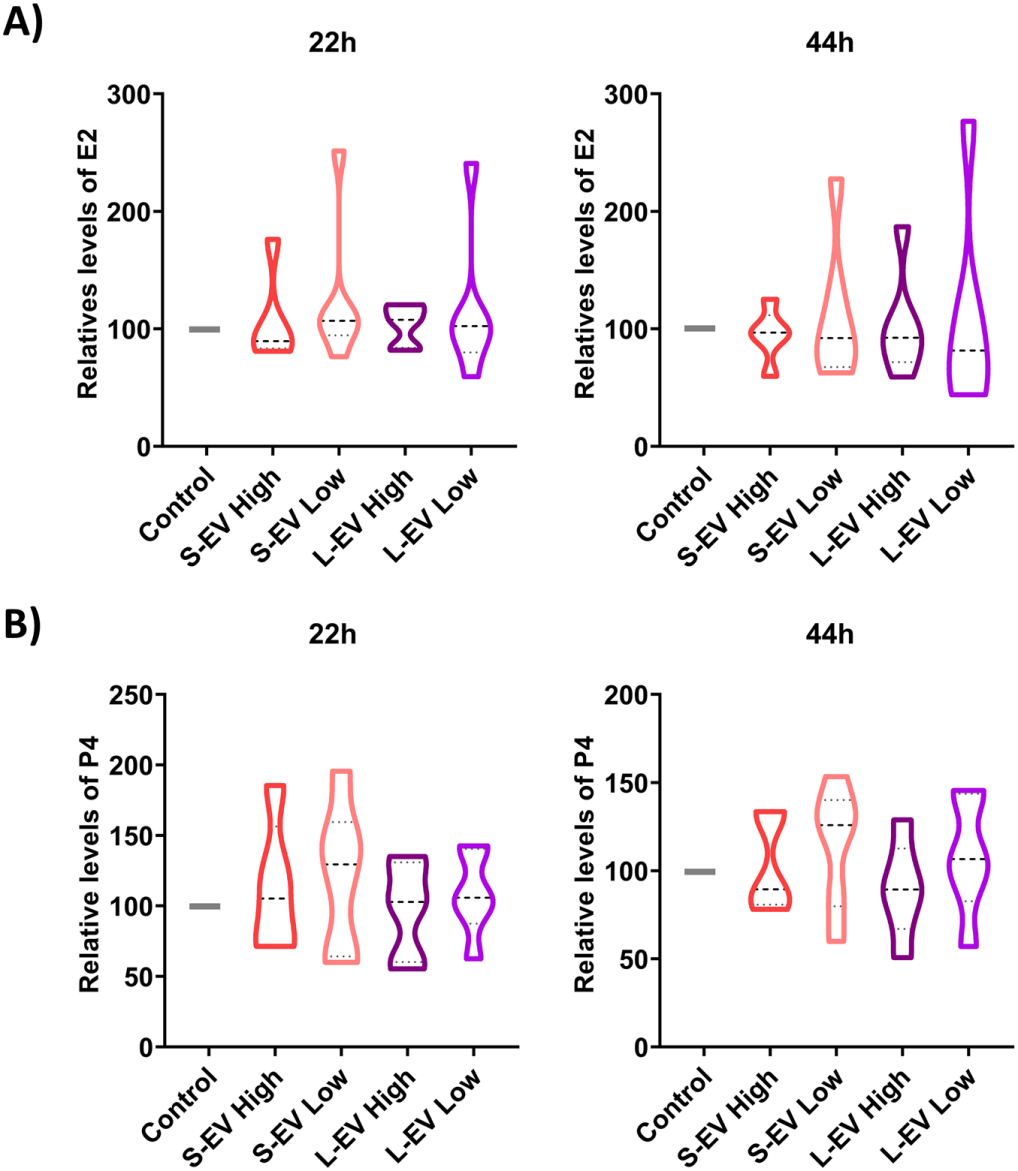
Violin plots representing relative levels of progesterone (P4) and estradiol-17β (E2) production by CCS in response to small- (SP-EV) or large-extracellular vesicles (L-EV) at two concentrations (Total protein concentration; Low: 0.1 mg/mL; High: 0.2 mg/mL). Secretion of steroid hormones was evaluated in the spent IVM media after 22 h and 44 h of IVM. Dashed line represents the median and dotted lines the 25–75% quartiles. This figure was created using GraphPad Prism version 8.2.1 (GraphPad Software, Inc., La Jolla, CA, USA; https://www.graphpad.com/).

In view of the qPCR results, the effect of the two SP-EV subsets on CCs steroidogenesis was further assessed by the quantification of steroids P4 and estradiol-17β (E2) in the IVM media spent after two days of culture of COCs. None of the SP-EVs concentrations tested influenced the production of E2 and P4 by CCs, regardless of the IVM period (22 or 44 h) (Fig. 4).

## Discussion

In this study, the interaction of two subsets of SP-EVs (S-EVs and L-EVs) with porcine COCs during IVM was reported for the first time. The results revealed that the two SP-EVs subsets were able to bind CCs during IVM, without affecting oocyte maturation, or apoptosis and cell proliferation pathways. Supplementation of COCs with L-EV during IVM was also seen to induce changes in the transcript levels of *HAS2*, *CYP11A1* and *HSD3B1* in CCs. Yet, no effect of SP-EVs supplementation on CCs steroidogenesis was found when steroid hormones (P4 and E2) were assessed in the spent IVM medium.

One of the greatest drawbacks that limits the EV-research field is the lack of standardisation on the isolation of EVs and their subsets^53^. While ultracentrifugation is still regarded as the gold standard, size exclusion chromatography (SEC)-based EV isolation is becoming increasingly popular^54^, allowing the isolation of a more functional and purer EV-population than that obtained by ultracentrifugation^55^. Herein, the EV-subsets were isolated from SP using a SEC-based procedure previously described by Barranco et al.^56^. Another limiting step in the functional studies of EVs is their characterisation. In accordance with Minimal information for studies of extracellular vesicles 2018 (MISEV 2018) guidelines^53^, multiple EV-characterisation was performed using complementary techniques to verify the phenotype, purity and functionality of the isolated SP-EV-subsets. The results of TEM, DLS, NTA and flow cytometry demonstrated that isolated SP-EV subsets were functional and in a high degree of purity, the latter proved by the reduced presence of lipoproteins contaminants, such as albumin.

Recent studies showed that SP-EVs bind sperm and regulate their function^24^. In addition, when they reach the female genital tract, either attached to the sperm plasma membrane or free, SP-EVs can interact with the endometrium and modulate the immune/inflammatory response and decidualisation^36–39^. The functional effect of SP-EVs on the female gamete, nevertheless, has been poorly investigated. Only a study conducted in rabbits reported that EVs isolated from prostate, epididymis and testis primary cultures were able to interact with female gametes during IVM^49^. As the biological activity of EVs relies on the ability of target cells to bind and integrate them^57^, the first experiment aimed to determine the uptake of the two SP-EVs subsets by COCs during IVM. Results demonstrated that S-EVs and L-EVs isolated from porcine SP were able to bind CCs, but not oocytes, during IVM. Similarly, Abumaghaid et al. (2022) examined the uptake of EV isolated from rabbit epididymal primary cultures by COCs, showing that EVs were able to bind CCs^49^. Several reports described the ability of EVs isolated from other reproductive biofluids, such as the follicular (Porcine^58,59^; Equine^60^; Bovine^61^) and oviductal ones (Canine^62^), to bind COCs. In agreement with the results showed herein, most of these works identified labeled EVs in CCs, but not in oocytes. Remarkably, although these studies used EVs released by different cell types, they concur with the current research in the failure of EV to bind the oocyte membrane. In contrast with this, Da Silveria et al. (2017) identified EVs isolated from bovine follicular fluid within the transzonal projections of CCs^60^. These findings suggest that: (1) the potential effect of EVs on the oocyte during IVM might be driven by the modulation of CCs physiology rather than through a direct effect on the oocyte; (2) EVs release their cargo within CCs, leading to a further transport of the molecules to the oocyte through gap junctions; or (3) EVs are transported by transzonal projections of CCs to the oocyte. Further research to confirm these hypotheses is needed.

Considering that the two SP-EV subsets were able to bind CCs, the second experiment focused on evaluating the potential effect of SP-EVs on CCs. To this end, specific signaling pathways in CCs were interrogated. The qPCR results evidenced that none of the two SP-EV subsets influenced the apoptosis of CCs. In addition, SP-EV subsets did not modify the relative expression levels of genes selected to evaluate cell proliferation or oocyte maturation, except for *HAS2*, which encodes Hyaluronan synthase 2, an enzyme essential for the synthesis of hyaluronic acid and, therefore, CCs expansion during oocyte maturation^63^. Whilst an effect of any of the SP-EVs subsets on oocyte nuclear maturation was observed at the end of IVM, these results could indicate that L-EV might influence CCs expansion. Yet, because no macroscopic differences in CCs expansion between the different experimental groups were detected in this study (data not shown), further research to investigate the effect of SP-EVs on CCs expansion is needed. This is of particular relevance if one takes into consideration that the EVs isolated from the primary culture medium of testis, epididymis and prostate enlarge the cumulus in rabbits^49^, and that exosome-like vesicles from follicular fluid also induce the expansion of CCs in porcine^58^. As most of the genes evaluated in this study did not appear to be influenced by SP-EVs, it could be that the EVs isolated from other reproductive biofluids have greater influence on the functional activity of CCs. This is the case of the EVs isolated from the follicular fluid, which upregulate the expression of genes related to cell proliferation and apoptosis in porcine CCs^59^, and those isolated from the oviductal fluid, which modulate the expression of miRNAs that target genes related to follicular growth, luteogenesis and steroidogenesis^62^. Considering that EVs deliver a specific cargo to trigger specific responses in target cells, it is reasonable to suggest that cargoes of EVs isolated from SP and female reproductive fluids are different. In support of this, Abumaghaid et al. (2022) found that the effect of EVs on the expression of genes related to ovarian function and oocyte maturation in CCs relies on the cell origin (epididymis, prostate and testis)^49^.

The potential effect of the two SP-EV subsets on CCs steroidogenesis was also investigated in this study. The expression of two steroidogenesis-related genes, *CYP11A1* and *HSD3B1*, was affected by L-EVs but not by S-EVs. These results concur with a previous work by Yuan et al.^59^, who reported that the EVs isolated from follicular fluid increases the expression of these two genes in porcine CCs. The fact that L-EVs, but not S-EVs, were found to influence the expression of these genes would suggest a specific function and cargo of each EV subset. In agreement with this possibility, research conducted in other mammalian species (human and sheep) has already demonstrated that the proteome profiles differ between SP-EV subsets^20,22,23,64^. On the other hand, *CYP11A1* gene encodes for Cytochrome P450 Family 11 Subfamily A Member 1, a mitochondrial enzyme that catalyzes the first and rate-limiting step of steroidogenesis, the conversion of cholesterol into pregnenolone^65,66^. Pregnenolone is the common precursor of the synthesis of steroid hormones including P4, testosterone, estrogen, or cortisol, among others. The *HSD3B1* encodes for Hydroxy-Delta-5-Steroid Dehydrogenase, 3 Beta- and Steroid Delta-Isomerase 1, an enzyme that is able to convert pregnenolone into P4 and also produce testosterone and estrogen precursors^67,68^. Based on the results of the current study, the L-EVs from SP could modulate these two pathways, and, thus, steroidogenesis in CCs. For this reason, whether changes in these genes ultimately influence P4 and E2 secretion by CCs was interrogated. Yet, neither S-EVs nor L-EV changed the pattern of P4 and E2 secretion by CCs, though P4 production was dramatically increased in all the experimental groups during the second 22 h of IVM, probably due to differentiation/luteinisation of CCs^69^. For this reason, because (i) the expression of steroidogenesis-related genes in CCs was modified by L-EV; (ii) P4 and E2 were unaffected by SP-EV; and (iii) steroid hormones secreted by COCs other than P4 and E2 have been proposed to contribute to oocyte maturation and CCs expansion in bovine^70^, it could be that SP-EV modulate the secretion of other steroid hormones in CCs. Further studies, nevertheless, are needed to address this hypothesis.

In conclusion, the research on SP-EV and their involvement in reproductive events is still in its infancy. The present study shows, for the first time in a mammalian species, the ability of SP-EV subsets to interact with COCs. Specifically, the two SP-EV-subsets were able to bind to CCs during IVM, with L-EV being able to modify the expression of genes involved in CCs expansion (*HAS2*) and steroidogenesis, in particular *CYP11A1* and *HSD3B1*. As none of the SP-EV subsets, however, exerted an effect on P4 and E2 secretion by CCs, oocyte maturation and the expression of other genes, it appears that SP-EV subsets do not have a dramatic influence on CCs function during IVM. The comparison of these results with other studies that demonstrated a positive effect of EVs from female reproductive fluids on CCs suggests that donor cells from which EV are released govern signalling pathways in targeted cells through the cargo of the EVs, which is likely to be specific of each type of EV. As the cargo of pig SP-EVs has not yet been unravelled, it is not possible to assert whether one or more molecules common to all EVs are involved in the response of CCs. It is also worth mentioning that four pathways were evaluated in the present study and, therefore, it cannot be discarded that SP-EV regulate other signalling pathways that could influence oocyte maturation, fertilisation and, ultimately, embryo development.

## Methods

### Ethic statement

As no animal was manipulated by the authors but rather the artificial insemination (AI)-centre provided ejaculates, and ovaries were collected from gilts slaughtered at an abattoir, no permission from an Ethics Committee was required.

### Reagents

Unless stated otherwise, all reagents used herein were of analytical grade and purchased from Merck (Darmstadt, Germany). Fluorochromes were acquired from Thermo Fisher Scientific (Waltham, MA, USA).

### Animals and ejaculates

Entire ejaculates (*n* = 5) were collected from healthy, mature fertile boars (one ejaculate per boar) housed in a commercial AI-centre (AIM Ibérica; Topigs Norsvin Spain SLU), with Spanish and European registration numbers ES300130640127 (August 2006) and ES13RS04P (July 2012), respectively. This AI-centre fulfilled the current Spanish and European legislation for commercialisation of pig semen, and animal health and welfare. All ejaculates complied with the thresholds of sperm quantity and quality for elaborating semen AI-doses (i.e., sperm concentration > 200 × 10^6^ sperm/mL, motile sperm > 70% and sperm with normal morphology > 75%). Immediately after collection, 10-mL samples from five ejaculates were centrifuged twice at 1,500 *xg* for 10 min (Rotofix 32A, Hettich Centrifuge UK, Newport Pagnell, Buckinghamshire, England, UK) at room temperature (RT) for harvesting SP. Then, SP samples were examined under a microscope (Eclipse E400; Nikon, Tokyo, Japan) to ensure they were sperm-free, and transported in an isolated container (5 °C) to the laboratory. Once in the lab, SP samples were mixed in one pool that was split into seven 4 mL-aliquots for further EVs isolation.

### Isolation of EV-subsets from SP

Two EVs-subsets (S-EVs and L-EVs) were isolated from SP following a previously described procedure^56^. Briefly, aliquots of SP were centrifuged at 3,200 *xg* and 4 °C for 15 min (Sorvall™ STR40, Thermo Fisher Scientific) to remove any cell debris. Supernatants were transferred into new tubes and centrifuged again at 20,000 *xg* and 4 °C for 30 min (Sorvall™ Legend™ Micro 21R, Thermo Fisher Scientific); the resulting pellets and supernatants were separately processed. Pellets (containing the larger EVs) were resuspended in 500 μL of 0.22-μm filtered PBS and stored (5 °C) until EVs isolation. Supernatants (2 mL; containing the smaller EVs) were diluted (1:2; v:v) in 0.22-μm filtered PBS, filtered (0.22 µm; Millex® Syringe Filters) and concentrated (Amicon® Ultra-4 mL centrifugal filter 10 kDa) by repeated centrifugations at 3,200* g* and 4 °C for 90 min. The resulting samples (~ 2 mL) were stored (5 °C) until EVs isolation. Isolation of EVs was carried out following a SEC-based procedure; 10 mL-columns were handmade using 12-filtration tubes stacked with Sepharose-CL2B® (10 mL). Prior to use, columns were washed with 60 mL of 0.22-µm filtered PBS. Then, samples were loaded onto a SEC-column followed by elution through 0.22-μm filtered PBS. Twenty sequential 500 µL-fractions were collected, and fractions 7 to 10 (enriched in EVs) were selected and mixed. The EV-samples resulting from 20,000 *xg* pellets and supernatants were named as L-EVs and S-EVs, respectively. The isolated EVs were stored at − 80 °C (EV-samples; Ultra Low Freezer; Haier Inc., Qingdao, China).

### Characterisation of SP-EV subsets

Isolated EVs were characterised using several and complementary analytic approaches, following MISEV (2018) guidelines^53^. Specifically, EVs were characterised in terms of (1) concentration and size distribution (by total protein concentration measurement, NTA and DLS); (2) morphology and size (by TEM); (3) EV-specific proteins (by flow cytometry); and (4) EV-purity (albumin content measurement by flow cytometry). Supplementary File 2 provides details of the EV-characterisation.

### Oocyte collection and in vitro maturation

Porcine ovaries were collected from pre‐pubertal gilts at a local slaughterhouse and transported to the laboratory at 37 °C in saline solution (0.9 w/v NaCl solution) within 2 h. COCs were aspirated from antral follicles (3–6 mm in diameter) through an 18‐gauge needle fixed to a 10‐mL syringe. Thereafter, intact COCs with more than three layers of compact CCs and with uniform cytoplasm were selected under a stereomicroscope and transferred into a petri dish (35 mm, Nunclon, Denmark) prefilled with 2 mL of PBS supplemented with BSA (0.4%). IVM was conducted following the procedure described by Spinaci et al. (2020)^71^. Briefly, COCs were washed three times in IVM medium (NCSU 37 supplemented with insulin (5 μg/mL), glutamine (1 mM), cysteine (0.57 mM), epidermal growth factor (10 ng/mL), β-mercaptoethanol (50 μM) and porcine follicular fluid (10%)). Groups of 40-COCs were transferred to a Nunc 4-well multidish prefilled with the same medium (400 μL) and cultured at 39 °C in a humidified atmosphere of 5% CO_2_ in air for 44 h. This 44 h-period of IVM was divided in two stages (22 h each). For the first 22 h of IVM, the IVM medium was supplemented with 1.0 mM db-cAMP and 0.12 IU/mL porcine FSH/LH (Pluset®, Calier, Italy). For the second period of 22 h, COCs were transferred to fresh IVM medium^72^. After the 44 h of IVM, oocytes were denuded by gentle repeated pipetting and then mounted on microscope slides, fixed in acetic acid/ethanol (1:3) for 24 h and stained with Lacmoid. Oocytes were observed under a phase contrast microscope to evaluate the meiotic stage achieved. Oocytes exhibiting a nuclear morphology corresponding to MII were considered mature.

### SP-EVs labelling

To examine the uptake of SP-EVs by COCs, SP-EV subsets were labelled with PKH67 Green Fluorescent Cell Linker kit (MIDI67), a green-fluorescent dye that labels membrane lipids and is widely used for visualisation of EVs uptake by cells^73^. The EVs were stained following the protocol described by Almiñana et al.^74^ with slight modifications. Briefly, each EV-sample (S-EV and L-EV; 25 µL) was mixed with diluent C (1 µL). Then, PKH67 (1 µL) was diluted in diluent C (100 µL), and the resulting volume was added to the previously prepared sample. After 5 min of incubation at room temperature, EV-depleted foetal bovine serum (FBS; 250 µL) was added to the mixture and incubated for 1 min to stop the labelling. This EV-depleted FSB was prepared the previous day by ultracentrifugation of heat inactivated FBS (ref. 10,500–056; Gibco, Thermo Fisher Scientific) at 100,000 *xg* and 4 °C for 16 h (Optima L-100 XP Ultracentrifuge using rotor SW55; Beckman Coulter, CA, USA). Two washes were made by filling 1-mL ultracentrifugation tubes (50 Ultra-Clear™ tubes; Beckman Coulter) with filtered PBS and subsequent ultracentrifugation at 100,000 *xg* and 4 °C for 30 min. The SP-EV labelled pellet was resuspended in IVM medium and stored at 4 °C until IVM was performed the next day following the aforementioned protocol.

### Quantitative real-time PCR (qPCR) analysis of gene expression in CCs

Changes in gene expression of CCs in response to SP-EV during IVM were analysed by qPCR. Separate signalling pathways were investigated using a total of nine genes: (i) cell apoptosis (*BCL2* and *BAX*); (ii) cell proliferation (*CCNB1*); (iii) oocyte maturation (*CX43*, *HAS2* and *SCD1*); and (iv) steroidogenesis (*CYP11A1*, *HSD3B1* and *CYP19A1*). All these candidate genes, including housekeeping *60S Ribosomal Protein L18* (*RPL19*), were selected on the basis of previous literature and were representative of each pathway analysed^63^.

Total RNA was extracted from CCs using RNeasy Mini kit (Qiagen; Hilden, Germany) following the manufacturer’s instructions. The quantity and purity of RNA was determined using the Epoch Microplate Spectrophotometer (BioTek; Winooski, VT, USA). For each sample, cDNA was prepared from the total RNA volume using the High-Capacity cDNA Reverse Transcription Kit (Thermo Fisher Scientific) according to the manufacturer’s instructions.

Gene specific primers were designed using Primer Blast software (https://www.ncbi.nlm.nih.gov/tools/primer-blast/; (Table 1). Primer efficiency was evaluated for all primers, and qPCRs of 1:2 dilutions (starting at 2.5 ng/µL and ending at 0.156 ng/µL) of a cDNA mix from a representative pool of cDNA CCs samples were analysed. Primers were considered as valid when they exhibited a single sharp peak in the melt curve and a standard curve with an efficiency value above 90%. The expression of these genes was individually evaluated in all samples using cDNA (0.625 ng), Fast SYBR™ Green Master Mix (10 μL), primers (1.2 μL of 5 µM) and nuclease-free water (5.1 μL). Reactions were developed in an Applied Biosystems 7500 Real-Time PCR Systems (Thermo Fisher Scientific) device, and thermo-cycling conditions were as follows: 1 cycle of holding stage at 50 °C for 2 min and 95 °C for 10 min; 40 cycles of cycling stage at 95 °C for 15 s and 60 °C for 1 min and, finally, 1 cycle of melting curve stage at 95 °C for 15 s, 60 °C for 1 min, 95 °C for 30 s and 60 °C for 15 s.

**Table 1:** Primer design.

**Gene**	**RefSeq (Sus scrofa)**	**Forward primer**	**Reverse primer**	**Tm (**°**C)**	**Amplicon size (bp)**
*RPL19*	XM_003131509.5	GGAAGGGTACTGCCAATGCT	GTGCTCCATGAGAATCCGCT	60	182
*BCL2*	XM_021099593.1	CAGCATGCGGCCTCTATTTG	CACTTATGGCCCAGATAGGCA	60	108
*BAX*	XM_021099593.1	GCCCTTTTGCTTCAGGGTTTC	CCAATGCGCTTGAGACACTC	60	131
*CCNB1*	NM_001170768.1	TGTGTGCCCAAGAAGATGCT	AGGGCGACCCAGACAAAAAT	60	189
*CX53*	NM_001244212.1	GGCAAGGTGAAAATGCGAGG	ATGGTTTTCTCCGTGGGACG	60	197
*CYP11A1*	XM_021098319.1	TGGTCCTGAACACGGAGGTA	GACATTGGTGATGGCTGAGAAC	60	146
*CYP19A1*	NM_214429.1	ATGGTGTCTGAAGTTGTGCCT	GACCTGGTATTGAAGATGTGTTTTT	60	103
*HAS2*	NM_214053.1	TGGAGCACCGGAAAATGAAAA	CGATGCAAAGAGCGACAGTT	60	89
*HSD3B1*	NM_001004049.2	ATTTCTCGGTGCCCAGGTTT	TGCTCTGGAGCTTAGAAAATTCC	60	181
*SCD1*	NM_213781.1	CGTCGCCACCTTTCTTCGTT	CCTCACCCACAGCTCCCAAT	60	146

RefSeq corresponds to Gene *NCBI* accession number. *PCR* conditions: melting temperature in °C (Tm) and amplicon size in bp.

Data were normalised using the comparative Livak Ct method (ΔΔCt method^75^). First, for each sample, the expression of the gene of interest was normalised to the expression of the housekeeping (*RPL19*), using the following formula: ΔCt = Ct_gene of interest_ − Ct_*RPL19*_. To calculate the ΔΔCt, results were scaled to the ΔCt of the control sample for each oocyte batch (no SP-EV) across all samples per target. The 2^−ΔΔCt^ values were used for the subsequent statistical analysis and results are shown as ΔΔCt.

### Determination of steroidogenesis in CCs

Spent IVM media from the two 22-h periods of COCs culture, either with the SP-EVs (S-EVs or L-EVs) or without them (control), were collected and centrifuged at 900 *xg* at room temperature for 5 min. The resulting supernatants were stored at − 20 °C until assayed for P4 and E2 by validated radioimmunoassays (RIA), as described by Galeati et al. ^69^. At the end of the 44-h IVM period, CCs were counted by a Thoma’s hemocytometer, after being released from oocytes by gentle pipetting. For P4, the intra- and inter-assay coefficients of variation were 4.1 and 7.3%, respectively; assay sensitivity was 3.8 pg/tube. For E2, the intra- and inter-assay coefficients of variation were 5.1 and 6.05%, respectively; the assay sensitivity was 1.1 pg/tube. Steroid concentrations are expressed as ng/10^6^ cells.

### Experimental design

**Figure 5:**
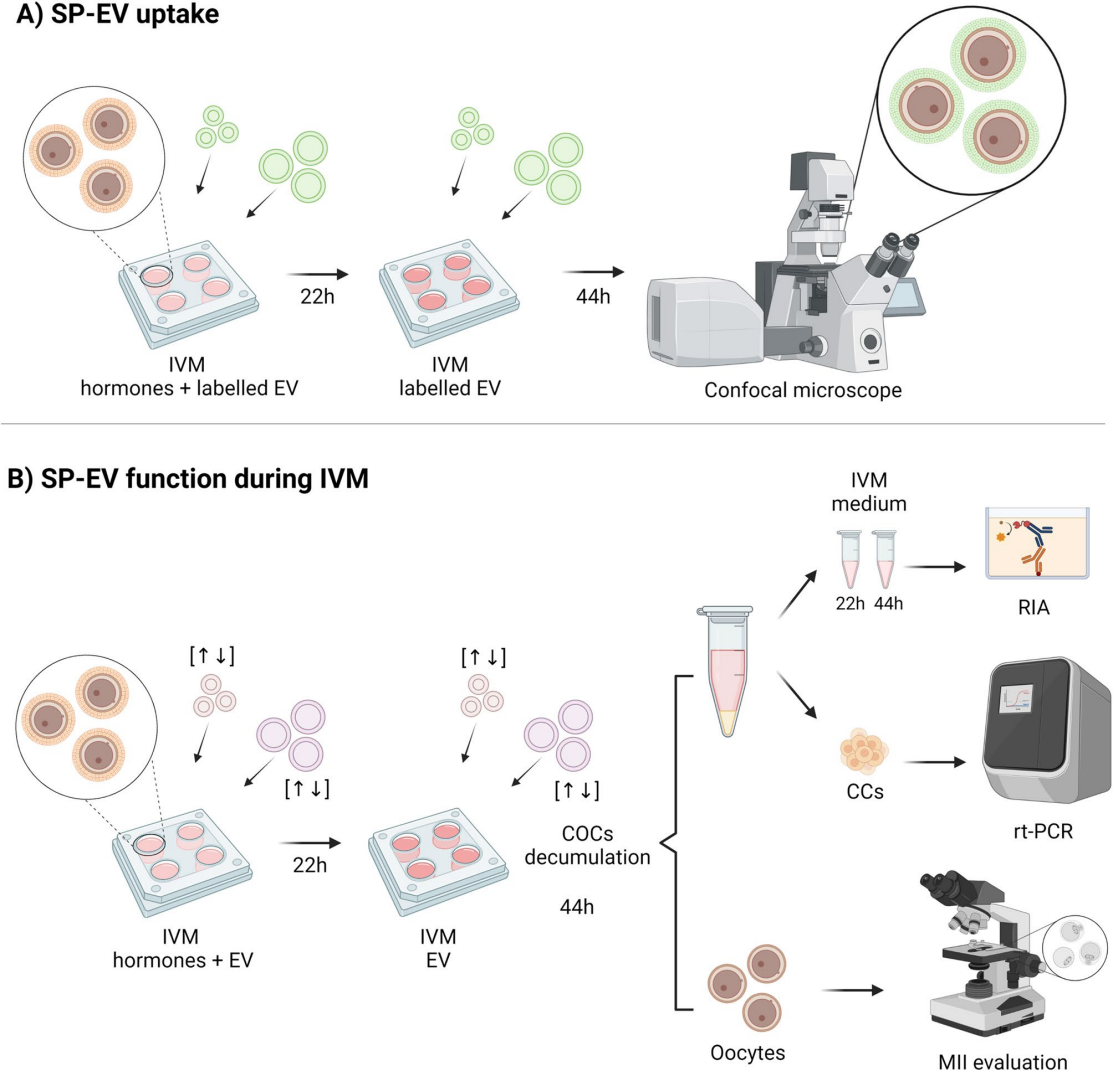
Experimental design. (**A**) Uptake of seminal extracellular vesicles (EV) by cumulus-oocyte complexes (COCs). COCs were in vitro matured (IVM) in the presence of labelled small- (S-EV) or large-extracellular vesicles (L-EV) for 44 h, and subsequently analysed using confocal microscopy. (**B**) Effect of SP-EV on COCs during IVM. COCs were in vitro matured in the presence of S-EV or L-EV; after 44 h, oocytes were fixed to evaluate the meiotic stage, and cumulus cells were retrieved to analyse their gene expression using quantitative real-time PCR. In addition, IVM medium was kept after 22 h and 44 h to analyse progesterone and estradiol-17β secretion using radioimmunoassay (RIA). The drawing was created with BioRender (https://biorender.com/).

A schematic overview of the experimental design is shown in Fig. 5. After SP-EV subsets (S-EVs and L-EVs) were accurately isolated and characterised, their effect on COCs was evaluated during the 44-h of IVM. For this purpose, two separate experiments were carried out in this study. The first experiment aimed to evaluate SP-EV (S-EVs and L-EVs) uptake by COCs during IVM. To this end, a total of 100 COCs were matured following the protocol described above in the presence or absence (negative control, PKH67 labelled PBS) of each PKH67 labeled SP-EV subset (S-EVs or L-EVs; 0.2 mg/mL total protein). At the end of IVM, COCs were imaged using a confocal laser scanning microscope (CLSM, Nikon A1R, Nikon Corp., Tokyo, Japan). Three replicates were performed. In each replicate, COCs from a different batch of ovaries were used. The second experiment was carried out to assess the effect of the two SP-EV subsets on COCs during IVM. In brief, COCs were incubated either with S-EVs or L-EVs at two different concentrations of total protein (Low: 0.1 mg/mL; High: 0.2 mg/mL, which were selected based on previous literature^74,76,77^) or without SP-EVs (control), during IVM. At the end of IVM (44-h), CCs were removed from the oocytes by repeated pipetting, and (1) oocytes were collected to evaluate the meiotic stage and (2) the IVM medium containing CCs was centrifuged at 1500 *xg* and room temperature for 5 min. Immediately after washing with PBS, CCs were stored at − 80° C and then used to assess gene expression (*BCL2, BAX, CCNB1, CX43, HAS2, SCD1, CYP11A1, HSD3B1, CYP19A1,* and *RPL19*) by qPCR. In parallel, spent medium of IVM was collected at the end of each 22-h IVM period to evaluate P4 and E2 secretion. Seven replicates were performed. In each replicate, 200 COCs were used (40 COCs per each group), which were collected from a different batch of ovaries (40–50). Hence, a total 1,400 COCs were used, which were collected from 280–350 ovaries.

### Statistical analysis

To avoid oocyte batch influence, data on the effect of SP-EV subsets on oocyte maturation, relative abundance of transcripts and hormone levels were normalised against the control of the same oocyte batch.

Results were analysed using IBM SPSS 27.0 (IBM Corp., Armonk, NY, USA). Data were first tested for normal distribution (Shapiro–Wilk test) and homogeneity of variances (Levene test), and linearly transformed through √*x* or arcsin √*x* when they did not fit with parametric assumptions. Following this, a linear mixed model (intra-subjects factor: time of IVM; inter-subjects factor: size and concentration of vesicles) or a one-way ANOVA (factor: size and concentration of vesicles) followed by post-hoc Bonferroni test for pair-wise comparisons were run. When linear transformations did not remedy normal distribution and/or homogeneity of variances, Friedman, Kruskal–Wallis and Mann–Whitney tests were used as non-parametric alternatives. The level of significance was set at *P* ≤ 0.05. Figures were created using GraphPad Prism version 8.2.1 (GraphPad Software, Inc., La Jolla, CA, USA; https://www.graphpad.com/) and Biorender (https://biorender.com/).

## Supplementary Information

Supplementary Information 1.

Supplementary Information 2.

## Data Availability

The datasets used and/or analysed during the current study are available from the corresponding authors on reasonable request.
