# Supplementary material for: Seminal extracellular vesicles subsets modulate gene expression in cumulus cells of porcine in vitro matured oocytes
Source: Sci Rep. 2022 Nov 9;12:19096. doi: 10.1038/s41598-022-22004-7 (PMC9646759; doi:10.1038/s41598-022-22004-7)
Supplement: Supplementary file 2 — Supplementary Information 2. [file 41598_2022_22004_MOESM2_ESM.docx]

**Supplementary File 2. Characterization of seminal extracellular vesicles (SP-EVs) subsets.** This characterization was conducted following Minimal information for studies of extracellular vesicles 2018 guidelines.

Total protein concentration was quantified in EV-samples using a commercially available kit (Micro BCA™ Protein Assay Kit; Thermo Scientific) and following the manufacturer’s instructions. Prior to total protein quantification, EV-samples (25 µL) were incubated (1:1, v/v) with lysis solution (0.1% Triton and 0.1% of sodium dodecyl sulphate) for 30 min at 37 °C under shaking. The absorbance was evaluated by a micro-plate reader (PowerWave XS; Bio-Tek Instruments, Winooski, Vermont, EEUU) at a wavelength of 562 nm.

Concentration and size distribution of isolated EVs were estimated using nanoparticle tracking analysis (NTA) and dynamic light scattering (DLS). NTA analysis was carried using a NanoSight LM10 (Malvern Instrument Ltd, Malvern, UK), equipped with a 405 nm laser and a scientific complementary metal–oxide–semiconductor camera. EV-samples were diluted in 0.22-μm filtered PBS to establish the particles number/field to 20-120 particles/frame. Data were analyzed with the NTA software (version 3.3.; Dev Build 3.3.104), with Min track Length, Blur and Max Jump Distance set to auto, and the detection threshold set to five. A total of five videos of 30 s at 30 frames/s were recorded for each EV-sample. DLS analysis was carried out using a Zetasizer Nano ZS-system (Malvern Panalytical, Malvern, United Kingdom) operating at 633 nm and recording the back scattered light at 173°. Briefly, each EV-sample (50 μL) was loaded into a 10 mm pathlength-cuvette. The light scattering was recorded for 150 s, performing three measurements per EV-sample. Dispersion Technology Software v.5.10 (Malvern Panalytical) was used to convert DLS signal intensity into particle size distribution, calculating EV diameter (nm) based on the peak maximum of the Gaussian function.

The morphology and size of EVs was estimated by TEM. For this purpose, the procedure described by Thery et al. (2006) was followed with slight modifications by using a JEOL JEM 1011 microscope at 80 kV (JEOL Ltd., Tokyo, Japan) (Théry et al., 2006). Briefly, each EV-sample (10 µL) was fixed in paraformaldehyde (2 %) for 30 min and set onto carbon-coated copper grids at room temperature for 15 min. After washing with 0.22-μm filtered PBS, samples were fixed with glutaraldehyde (1 %) at room temperature for 5 min. Then, samples were washed with distilled water and contrasted in uranyl-acetate (1 %), soaked in methylcellulose (0.5 %) and dried at room temperature (RT). The diameter of EVs was assessed using the ImageJ 1.41 software (National Institutes of Health, USA).

Identification of EV-specific proteins was performed using a high-resolution flow cytometer (CytoFLEX S; Beckman Coulter, Life Sciences Division Headquarters, Indianapolis, USA) following the procedure described by Barranco et al. (2022) and the ISEV recommendations (MIFlowCyt-EV (Welsh et al., 2020)). The proteins identified were transmembrane glycoprotein CD44, HSP90β, and albumin (one of the major components of non-EV co-isolated structures). First, EV-samples (10 μL) were incubated with CellTrace ™ CFSE (Thermo Fisher Scientific, Waltham, Massachusetts, USA) to differentiate intact EVs from membrane fragments. Following this, EV-samples were split into three aliquots and incubated separately with anti-HSP90β-PE (ADI-SPA-844PE-050, Enzo Life Sciences, Farmingdale, NY, USA), anti-CD44-FITC (MCA4703F, Bio-Rad, Hercules, California, USA) or anti-Albumin-FITC (CLFAG16140, Cedarlane, Burlington, Canada, USA) at room temperature for 30 min. Samples were subsequently diluted in 0.1 μm-PBS filtered to a final volume of 300 μL. Data were recorded using CytoFLEX S, provided with red (638 nm), yellow (561 nm), blue (488 nm), and violet (405 nm) lasers. Distilled water (0.1-μm filtered) was used as sheath fluid. A commercial calibration kit (Nanobead Calibration Kit, Bang Laboratories Inc., Technologies Drive Fisher, Indiana, USA), equipped with 50 and 100 nm-fluorescent microspheres, was used to verify the flow cytometer performance and to set the EV gate. Optic configuration was established to use SSC information from 405 nm laser (Violet-SSC-A). FSC and Violet-SSC-A and the fluorescence channels were acquired in linear scale but displayed in logarithmic scale. To ensure background noise removal and cytometer clean status, PBS alone (0.1 µm-filtered) was analysed before each EV-sample. Low-flow rate setting (10 µL/min) was established and 10 × 10^3^ events were acquired for each sample.

**References**

Barranco, I., Sánchez-López, C., Marcilla, A., Bucci, D., Tamanini, C., and Roca, J. (2022). A protocol for isolation of extracellular vesicle-subtypes from pig seminal plasma. *Reprod. Domest. Anim.* 57, 127.

Théry, C., Amigorena, S., Raposo, G., and Clayton, A. (2006). Isolation and characterization of exosomes from cell culture supernatants and biological fluids. *Curr. Protoc. cell Biol.* Chapter 3, Unit 3.22.

Welsh, J. A., Van Der Pol, E., Arkesteijn, G. J. A., Bremer, M., Brisson, A., Coumans, F., et al. (2020). MIFlowCyt-EV: a framework for standardized reporting of extracellular vesicle flow cytometry experiments. *J. Extracell. vesicles* 9, 1713526.
